# Editorial: New and advanced mechanistic insights into the influences of the infant gut microbiota on human health and disease

**DOI:** 10.3389/fmicb.2024.1507950

**Published:** 2024-11-26

**Authors:** Ran Wang, Renqiang Yu

**Affiliations:** ^1^Department of Neonatology, Affiliated Women's Hospital of Jiangnan University, Wuxi Maternity and Child Health Care Hospital, Wuxi, China; ^2^Department of Public Health and Preventive Medicine, Wuxi School of Medicine, Jiangnan University, Wuxi, China

**Keywords:** gut microbiota, probiotic, newborn infant, gut inflammation, dysbiosis

## 1 Introduction

The human gut microbiota plays a crucial role in both health and disease. These microbes provide essential nutrients to the host, offer colonization resistance against pathogens, and regulate the immune system functions. In early life, the gut microbiota undergoes significant changes influenced by environmental factors and immune system maturation, potentially influencing health throughout an individual's lifetime. Although bacterial colonization may begin *in utero*, the initial weeks postpartum represent a critical period for the establishment of the gut microbes. The infant gut microbiota exerts a lasting impact on subsequent health. Early perturbations in the gut microbiome can result in long-term health consequences, increasing the risk of acquiring chronic metabolic disorders such as diabetes and obesity (Lv et al., 2022). Recent studies have found that the microbiome of the preterm infant presents simple microbial communities and exposed to a consistent diet under controlled conditions. Due to physiological immaturity of the gut environment, the development of the gut microbial community in premature infants may be disrupted which may adversely affect the growth and health of premature infants. Premature infants are also more likely to develop bronchopulmonary dysplasia (BPD), necrotizing enterocolitis (NEC) and late-onset sepsis (LOS), which may be related to shifts in the composition of the gut microbiome (Tirone et al., 2019).

This Research Topic includes 22 articles highlighting the enduring effects of the infant gut microbiota, examining its relationship with gastrointestinal, respiratory, allergic, and even neurological disorders, along with factors influencing microbiota colonization.

## 2 Gastrointestinal disorders

The first 1,000 days after birth are critical to the development of gut microbiome. During this period, the gut microbiota of premature infants shows reduced diversity and delayed maturation as compared to the term infants. This delayed in maturity makes preterm vulnerable to several diseases such as NEC, a inflammatory severe gastrointestinal disease in preterm infants. Studies suggest that NEC development may be linked to the overgrowth of specific pathogenic microbes or a deficiency in protective microbiota. Further studies are needed to elucidate the causal relationship between the gut microbiome and NEC and to develop targeted prevention and treatment strategies based on these findings. The role of human breast milk microbiota in shaping the gut microbiome of preterm infants may offer useful insights (Zeng et al.). Therefore, finding new biomarkers in the gut microbiota is crucial for early prediction of NEC. A cohort study of preterm infants identified increased levels of *Streptococcus salivarius and Rothia mucilaginosa* in NEC subjects, while reduced abundance of *Bifidobacterium animalis* subsp. *lactis* and lower levels of short chain fatty acids (SCFA), acetate, propionate, and butyrate, suggesting their potential application as NEC biomarkers (Liu X.-C. et al.). Some researchers have attempted to use *Bifidobacterium longum BAA-2573* to intervene in colitis and have found beneficial effects linked to an increase in SCFA (Lin et al.). *Lactobacillus paracasei BNCC345679* can also reduce colitis symptoms by restoring the diversity of the intestinal microbiota, especially increasing the abundance of beneficial bacteria such as *Lactobacillus* sp and *Akkermansia* sp (Ahmad et al.). In addition to NEC, ulcerative colitis (UC) may also benefit from microbiota modulation. UC is a global public health problem, with a rising incidence, especially among children. Dysbiosis of the gut microbiota is considered the main cause of chronic intestinal inflammation. So probiotics have emerged as adjunctive therapy for UC in recent years, aiding in microbiota restructuring, enhancing intestinal barriers, regulating immune responses and reducing inflammation. However, its safety and clinical application still need further research (Huang et al.). In recent years, the gut-brain axis has played an increasingly important role in the treatment of nervous system diseases. Epilepsy is a chronic nervous system disease. Clinical studies in infants with epilepsy accompanied by diarrhea have found that *Bifidobacterium* may be downregulated, suggesting it could serve as a potential intestinal flora marker and aid in developing new diagnostic and treatment strategies for epilepsy (Liu T. et al.). In addition, some researchers verified the association between intestinal flora and Hirschsprung disease (HSCR) through a public database using a two-sample Mendelian randomization method (MR). Two-sample MR is used to assess the causal relationship between exposure and outcome. Forward MR analyzes the impact of exposure on the outcome, while reverse MR examines the influence of the outcome on the exposure. In the forward MR analysis, *Eggerthella* was found to be associated with an increased risk of HSCR, while *Peptococcus, Ruminococcus and Praprevotella* were associated with a reduced risk of HSCR. Reverse MR analysis showed that HSCR is the risk factor for *Eggerthella*, which could provide a new strategy for HSCR treatment (Liu et al.). In addition to the above clinical and preclinical studies, the Research Topic also emphasizes the importance of some clinical techniques, such as the high sensitivity and specificity of abdominal ultrasound in diagnosing NEC perforations, which helps to detect severely ischemic or necrotic intestinal segments earlier, thereby possibly reducing the morbidity and mortality of NEC (Chen et al.).

## 3 Respiratory diseases

BPD is a severe chronic lung disease common in premature infants. Clinical studies have shown that children with BPD have lower intestinal microbial diversity than healthy individuals, and there are significant differences in the intestinal microbiota structure between the two groups. Children with BPD have a higher relative abundance of *Proteobacteria*, while *Firmicutes* have a lower relative abundance (Zhang et al.). Therefore, gut microbial modulations of patients with BPD is also a new strategy for the treatment of BPD. There is a significant correlation between intestinal microorganisms in children and allergic rhinitis (AR), and changes in intestinal microorganisms are significantly correlated with clinical symptoms in children with AR, such as nasal symptom scores, eosinophil count, total immunoglobulin E level, etc. This indicates that the enteropulmonary axis is expected to become a new strategy for the treatment of AR (Zhang et al.). The gut-lung axis is an important mechanism affecting gut and lung immunity, but few studies have examined the correlation between gut and pharyngeal microbial communities in early newborns. Clinical studies have shown that the initial colonization of microbiota is closely related to the ecological niche environment in the intestine and oropharynx, with their core microbiota being closely correlated. However, the intestinal microbiota was more predictive than the oropharyngeal microbiota in transcription, metabolism, cell motility, cellular processes and signaling, and organismal system function in the KEGG pathway (Wang et al.). In addition to the gut-brain axis and the gut-lung axis, a study mentioned the gut-spinal axis for the first time, emphasizing that the intestinal microbiota affects the host's metabolic, immune and endocrine environment through complex mechanisms, and has a close interaction with spinal degenerative diseases (SDD). SDD can be treated by regulating the intestinal microbiota (Morimoto et al.). The Research Topic also includes research on oral microorganisms in infants and young children, such as the dynamic changes of oral flora in newborns. The oral microbiome, associated with both oral disease and systemic disease, is in dynamic status throughout the life, and many factors including maternal microbiomes could impact the oral microbiome. While fewer studies have been conducted to study the characteristics of the oral microbiome in neonates and the associated maternal factors. The oral microbiota of newborns changes significantly within the first 4 days of birth. Under normal conditions many bacteria that found in vagina, skin and environment, disappear in the mouth over time. Meanwhile, *Staphylococcus epidermidis* RP62A phage SP-beta, predominate bacterium in maternal skin microbiome and *Streptococcus* unclassified, important bacterium in vaginal microbiome, increased in neonatal oral microbiome over the time, and the composition of the oral microbiome in the neonates was more similar to that of the milk microbiome in their mothers. Moreover, we found that the changes in the predominant bacteria in the neonates were in line with those in the neonates exposed to the environment. This study have helped subsequent researchers better understand the role of the maternal environment in the maturation of the neonatal oral microbiota and may have an impact on future oral health and disease prevention strategies (Guo et al.).

## 4 Factors affecting the intestinal microbiota

Infants 'intestinal microbiota is mainly colonized after birth. With age, microbial diversity increases and stabilizes at the age of 3–5 years, representing adult like microbiota. Prenatal factors, including the mother's diet during pregnancy, antibiotic use, delivery method, and gestational age, can affect the infant's intestinal microbial colonization. These factors may affect the infant's intestinal microbial colonization by affecting the mother's intestinal microbiota. For example, the gut microbial colonization of preterm infants remain highly at the different time points after birth, *Exiguobacterium, Acinetobacter*, and *Citrobacter* showed reduced abundance with the advancement of age, while the bacterial groups of *Enterococcus* (*Klebsiella* and *Escherichia coli*) gradually increased and became the major portion of the gut microbiota during the development of gut microbiome in preterm infants at the age of 42 days. This offers a new perspective on targeting specific bacteria at different postnatal time points for the treatment of premature infants (Khan et al.). Postpartum feeding methods, such as breastfeeding or formula feeding, antibiotic use in infants and young children, and environmental factors, socioeconomic conditions and dietary patterns can also affect the early gut microbial colonization (Suárez-Martínez et al.). For example, long-chain fatty acids in breast milk are highly correlated with edible oils in the mother's diet and may further affect the abundance of specific microorganisms in the intestines of infants, such as regulating the abundance of *Lactobacillus rhamnosus, Lactobacillus fermentum*, and *Lactobacillus paracasei* in the intestines of infants. Therefore, special attention should be paid to dietary habits during pregnancy and lactation (Xi et al.). Studies have found that supplementation of probiotics can increase the relative abundance of *Enterococcus* and *Enterobacter* and reduce the relative abundance of *Escherichia* and *Clostridium* in premature infants, providing new ideas and strategies for the prevention and treatment of neonatal related diseases including NEC and LOS (Yang et al.).

Existing research on gut microbiota has some limitations, such as small sample size, limited longitudinal research on the long-term health impact of infants' intestinal microbiota. As a result, in-depth insights into these long term effects may be insufficient, and long-term follow-up studies are needed to explore the impact on health in adulthood. This includes examining how intestinal microbiota colonization in infants and young children may influence the risk of common chronic diseases later in life. Greater attention should be given to these areas in future research.
